# Norovirus Contamination Levels in Ground Water Treatment Systems Used for Food-Catering Facilities in South Korea

**DOI:** 10.3390/v5071646

**Published:** 2013-07-02

**Authors:** Bo-Ram Lee, Sung-Geun Lee, Jong-Hyun Park, Kwang-Yup Kim, Sang-Ryeol Ryu, Ok-Jae Rhee, Jeong-Woong Park, Jeong-Su Lee, Soon-Young Paik

**Affiliations:** 1 Department of Microbiology, College of Medicine, the Catholic University of Korea, Seoul 137-701, Korea; E-Mail: borami0828@catholic.ac.kr; 2 Korea Zoonosis Research Institute, Chonbuk National University, Jeonju 561-756, Korea; E-Mail: shocklsg@hanmail.net; 3 Department of Food Science and Biotechnology, Kyungwon University, Seongnam 461-701, Korea; E-Mail: p5062@kyungwon.ac.kr; 4 Department of Food Science and Technology, Chungbuk National University Korea, Chongju 361-763, Korea; E-Mail: kimky@chungbuk.ac.kr; 5 Department of Food Science and Biotechnology, Seoul National University Korea, Seoul 151-742, Korea; E-Mail: sangyu@snu.ac.kr; 6 DK EcoV Environmental Microbiology Lab, Biotechnology Business Incubating Center, Dankook University, Chungnam 330-714, Korea; E-Mail: ojrh22@nate.com; 7 Sanigen Co. Ltd., Juan-dong, Gwacheon-si, Gyeonggi-do 427-070, Korea; E-Mail: withgaia@sanigen.co.kr; 8 Food Microbiology Division, Food Safety Evaluation Department, National Institute of Food and Drug Safety Evaluation, Osong 363-700, Korea; E-Mail: djsimson77@korea.kr

**Keywords:** norovirus, groundwater, nationwide, genotype

## Abstract

This study aimed to inspect norovirus contamination of groundwater treatment systems used in food-catering facilities located in South Korea. A nationwide study was performed in 2010. Water samples were collected and, for the analysis of water quality, the temperature, pH, turbidity, and residual chlorine content were assessed. To detect norovirus genotypes GI and GII, RT-PCR and semi-nested PCR were performed with specific NV-GI and NV-GII primer sets, respectively. The PCR products amplified from the detected strains were then subjected to sequence analyses. Of 1,090 samples collected in 2010, seven (0.64%) were found to be norovirus-positive. Specifically, one norovirus strain was identified to have the GI-6 genotype, and six GII strains had the GII, GII-3, GII-4, and GII-17 genotypes. The very low detection rate of norovirus most likely reflects the preventative measures used. However, this virus can spread rapidly from person to person in crowded, enclosed places such as the schools investigated in this study. To promote better public health and sanitary conditions, it is necessary to periodically monitor noroviruses that frequently cause epidemic food poisoning in South Korea.

## 1. Introduction

Noroviruses (NoVs), the most common cause of epidemic food and waterborne viral gastroenteritis [1], are recognized as the most prevalent cause of severe epidemics of acute nonbacterial gastroenteritis worldwide, which is considered a significant public health burden [2]. 

NoVs are a members of the *Caliciviridae* family and have a positive-sense, single-stranded RNA (7.4–8.3 kb) [3]. NoVs can genetically be classified into five different genogroups (GI to GV), which can be further subdivided into genetic clusters or genotypes. GI, GII and GIV infect humans, whereas GIII infects bovine species, and GV infects mice. According to a recent study, GII also infects porcine species, and GIV infects both feline and canine species. Additonally, NoV has recently been isolated in lion [3,4,5].

The Centers for Disease Control and Prevention estimates that 900,000 clinic visits by children each year in industrialized countries occur as a result of NoV outbreaks, and estimates 640,000 hospitalizations due to diarrhea. In developing countries, NoVs have also been estimated to cause more than 200,000 deaths among children under the age of five every year [6]. In South Korea, NoV-related viral gastroenteritis has been a major public health problem since 2005, when the virus was reported for the first time [7,8].

The World Health Organization defined foodborne diseases as an infectious disease caused by ingesting contaminated food or water, in 1999 [9]. In Sweden, numerous recent NoV outbreaks due to contaminated water and food, such as by eating shellfish and raw salad, have caused foodborne and waterborne gastroenteritis [10]. 

NoVs are transmitted by the fecal-oral route through person-to-person or through feces contaminated food and water [11]. The occurrence rate of NoVs in groundwater has been reported to be approximately 8%–21% worldwide [12]. In particular, numerous outbreaks of gastroenteritis were caused by NoV contamination in drinking water [13]. Recently, the number of patients with foodborne diseases has increased because of the increase in the incidence of eating out and in food-catering facilities in South Korea [14]. A number of outbreaks and sporadic cases have been caused by waterborne and foodborne NoVs in South Korea. In particular, during 2007–2009, NoV was responsible for 16.5% of the waterborne and foodborne disease outbreaks reported in South Korea [15]. Waterborne outbreaks of NoV-associated acute gastroenteritis have been frequently reported worldwide [7,16,17,18]. Specifically, a number of waterborne NoV outbreaks have been documented to stem from contaminated drinking water [19,20,21,22], recreational water [23,24], and groundwater [7,25].

Furthermore, food-poisoning outbreaks in South Korea frequently involved NoV contamination in treated groundwater that is used for food-catering facilities. The aim of this study was therefore to survey NoVs in the groundwater which was treated by groundwater treatment systems, in food catering facilities located in South Korea.

## 2. Experimental Section

### 2.1. Collection and Processing of Water Samples

Groundwater samples which were treated by groundwater treatment systems were collected from 1,090 sites, selected by the Korea Food & Drug Administration, located in 8 provinces (Chungcheongnam-do, Chungcheonbuk-do, Jeollanam-do, Jeollabuk-do, Gyeongsangnam-do, Gyeongsangbuk-do, Gangwon-do, and Jeju-do) of South Korea in 2010; the 1,090 samples were obtained from the Waterborne Virus Bank [26].

Samples of 500 to 2,345 L were collected, depending on water turbidity; the latter ranged from 0.01 to 6.4 nephelometric turbidity units. All samples collected using a filter apparatus with a 1-MDS filter (ZetaPor Virosorp, Cuno, Research Parkway, Meriden, CT, USA) were eluted and further concentrated for subsequent NoV assays. Briefly, the sampled filter was subjected to elution by 1.5% beef extract and 0.05 M glycine (pH 9.5). The cartridge housing was filled and allowed to be in contact with elution buffer for 30 min. Then pressurized nitrogen gas was used to force the eluent out. The eluent was subjected to acid precipitation with 1 M HCl. The precipitate was centrifuged at 2,500 ×g at 4 °C for 15 min. The pellet was completely dissolved using 20 mL 0.15 M sodium phosphate (Na2HPO4•7H2O, pH 9.0–9.5). The suspension was centrifuged at 7,500 ×g at 4 °C for 10 min and the supernatant was carefully collected using a pipette. The processed eluent was adjusted to a neutral pH (7.0–7.5) with 1 M HCl. The sample was filtered through a 0.45-μm pore size syringe filter to remove non-viral organisms and stored at −70 °C until analysis.

### 2.2. Examination of Water Quality by Analyzing Physical-Chemical Parameters

The water temperature and pH were measured using portable electrode-carrying devices (PH-208; Lutron Electronic, Taipei, Taiwan). Residual chlorine and turbidity were measured with the HI 95701C Photometer and the HI 93703 Portable Microprocessor Turbidity Meter, respectively (HANNA Instruments, Woonsocket, RI, USA).

### 2.3. NoV Detection

Water samples processed with the 1-MDS filters as described above comprised a final eluate of 20 mL. Viral RNA was extracted from 140 µL of these eluates with the QIAamp Viral RNA Mini Kit (Qiagen, Hilden, Germany), according to the manufacturer’s protocol to obtain a final volume of 60 µL. 

Reverse transcription-PCR (RT-PCR) was conducted using the One Step RT-PCR Kit (iNtRON Biotechnology, Seoul, Korea). To detect NoV genotypes, semi-nested RT-PCR amplification was performed with previously described primer sets (NV-GIF1M/NV-GIR1M for NoV GI; and NV‑GIIF1M/NV-GIIR1M for NoV GII; Table 1). We used 5 µL viral RNA as template and 20 µL of the pre-mixed kit solution. Previously reported size-distinguishable products were used as the positive control [27]. PCR reactions were carried out in the S1000 Thermal Cycler (Bio-Rad, Foster, CA, USA) according to the following protocol: an initial RT step at 45 °C for 30 min, a PCR-activation step at 94 °C for 5 min, followed by 35 cycles each consisting of 94 °C for 30 s, 55 °C for 30 s, and 72 °C for 90 s, and then a final extension step at 72 °C for 7 min. Then, 2 µL of the abovementioned products was used as the templates for nested PCR, along with 48 µL of the Maxim PCR Premix Kit (iNtRON Biotechnology, Seoul, Korea) and specific primer sets (NV-GIF2/NV-GIR1M for NoV GI; and NV‑GIIF3M/NV-GIIR1M for NoV GII; Table 1). Nested PCR conditions were as follows: 94 °C for 5 min, then 25 cycles each consisting of 94 °C for 30 s, 55 °C for 30 s, and 72 °C for 90 s; followed by a final extension step at 72 °C for 10 min. PCR products were electrophoresed on 1.5% agarose gels, stained with ethidium bromide, and visualized under UV light. Finally, PCR products were directly sequence analyzed (Cosmogentech, Ltd, Seoul, South Korea) using the forward and reverse primers.

### 2.4. Statistical Analysis

Correlations between the NoV detection rate and water quality (physical-chemical parameters) were analyzed by the Chi-square test. 

### 2.5. Genotyping and Phylogenetic Analysis

For genotyping of sequenced products, the sequences were compared to those in the GenBank database using the NCBI BLAST search program. To confirm the genotype of NoV, phylogenetic analysis was performed and the phylogenetic trees were obtained using Clustal W and neighbor-joining methods with DNAStar version 5.07 software [28]. 

## 3. Results

### 3.1. Location and Seasonal Pattern of NoVs

In this study, among the 1,090 samples collected in 2010, seven (0.64%) were positive for NoVs. These seven water samples in which NoVs were detected were acquired from the following two provinces: one from a northeastern province and six from southwestern provinces. One case was detected in April, two cases in June, two cases in August, and two cases in October. The water sample collected on June 13, 2010, was only used for cleaning, while the other six positive samples were from water used for food preparation, drinking, and cleaning (Table 2).

### 3.2. Genotyping and Phylogenetic Analysis of NoVs

The results of sequence and phylogenetic analyses showed that the NV-GI primer sets were able to detect the GI genotype (GI-6) in one of the samples collected in 2010. The NV-GII primer sets were capable of detecting GII, GII-3, GII-4 and GII-17 genotypes in 1, 1, 2, and 2 of these samples respectively.

**Table 1:** Primers used for nested RT-PCR assays.

Genogroup	Region, and size (bp)	Primer/polarity	Sequence (5'-3')^d^	Position
**I**	Capsid (313)	NV-GIF1M/forward ^a^ NV-GIF2/forward ^b^ NV-GIR1M/reverse ^c^	CTG CCC GAA TTY GTA AAT GAT GAT ATG ATG ATG GCG TCT AAG GAC GC CCA ACC CAR CCA TTR TAC ATY TG	5342–5365 ^e^ 5358–5380 ^e^ 5649–5671 ^e^
**II**	Capsid (310)	NV-GIIF1M/forward ^a^ NV-GIIF3M/forward ^b^ NV-GIIR1M/reverse ^c^	GGG AGG GCG ATC GCA ATC T TTG TGA ATG AAG ATG GCG TCG ART CCR CCI GCA TRI CCR TTR TAC AT	5049–5067 ^f^ 5079–5102 ^f^ 5367–5389 ^f^

^a^ Primers for first PCR. ^b^ Primers for semi-nested PCR. ^c^ Primers for first and semi-nested PCR. ^d^ Degenerate position Y:C/T, R:A/G, I:Inosine. ^e^ Norwalk virus (GenBank accession no. M87661), a GI reference strain. ^f^ Lordsdale virus (GenBank accession no. X86557), a GII reference strain.

**Table 2:** Water quality (physical-chemical parameters) and microorganism data of NoV-contaminated samples.

Strainsname	Sampling date (day-m-y)	Sampling site/Settings	Use of groundwater	Amount of attainable water (L)	Temp (°C)	Turbidity (NTU)	pH	Residualchlorine (ppm)	Faculty capacity (person)	No. employees	No. foodhandlers	Max. no. people at once	NoV genotype
2010042241	22-Apr-2010	Chungcheongnam-do/ Elementary school	Food preparation, Drinking, Cleaning	800	15.2	0.39	7.7	0	166	2	2	60	II-4
2010060371	03-Jun-2010	Jeollabuk-do/ Elementary school	Food preparation, Drinking, Cleaning	800	17.8	0.3	6.52	0	217	3	2	94	II-4
2010071392	13-Jul-2010	Chungcheongnam-do/ Elementary school	Cleaning	580	17.5	0	3.8	0	178	4	3	75	II-3
2010081812	18-Aug-2010	Gangwon-do/ Elementary school	Food preparation, Drinking, Cleaning	602	22.3	0.07	6.7	0	60	2	2	40	I-6
2010082761	27-Aug-2010	Jeollanam-do/ Elementary school	Food preparation, Drinking, Cleaning	800	21.0	0.34	6.59	0	473	9	8	743	II-17
2010100772	07-Oct-2010	Jeollanam-do/ Middle school	Food preparation, Drinking, Cleaning	800	18.4	0.27	7.32	0	180	3	2	101	II-17
2010101112	11-Oct-2010	Chungcheongbuk-do/ Elementary school	Food preparation, Drinking, Cleaning	500	18.2	0	7.9	0	1272	21	2	66	II

**Figure 1:**
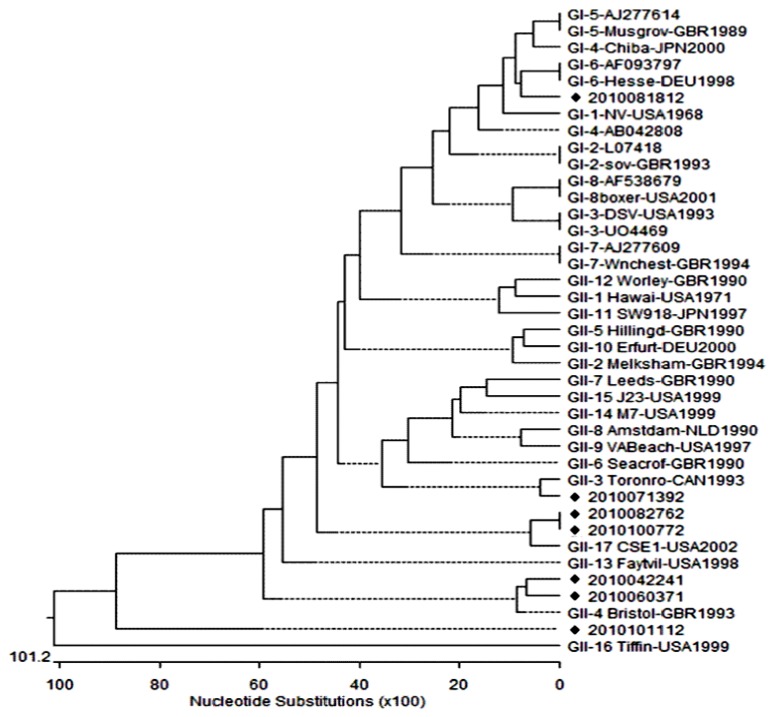
Phylogenetic trees constructed with the partial nucleotide sequences of norovirus capsid genes of strains isolated in South Korea in 2010.

A phylogenetic tree was constructed using the GI and GII genogroup reference strains, using the partial capsid region sequences (see Figure 1). Sequence and phylogenetic analysis showed that strain 2010081812 was identified as being GI-6, while the six strains were identified as GII (strains 2010042241, 2010060371, 2010071392, 2010082761, 2010100772, and 2010101112) were analyzed as being GII, GII-3, GII-4 and GII-7. Sequence analysis revealed that strain 2010081812 shared the greatest identity (85.7%) with AF-093797 and Hesse-DEU1998 strains, which had been isolated in Germany. Strain 2010071392 was found to be related most closely to Toronto-CAN 1993 (93.1%), whereas the 2010042241 and 2010060371 strains were clustered into the GII-4 genotype with 96.5% and 83.8% identity, respectively. In addition, the 2010082761 and 2010100772 strains showed 89.6% sequence identity to the GII-17 genotype.

## 4. Discussion

This study is the first study worldwide to focus on NoV contamination in treated groundwater used in catering facilities. In South Korea, most of the food-catering facilities that use groundwater for preparing food have frequently reported food-poisoning caused by NoV [15]. Because the incidence of food poisoning caused by NoV is increasing, monitoring of NoV contamination of water in the environment is an essential preventative measure. 

The number of positive samples was low (7 out of 1,090); thus, there were limitations in statistical analyses for correlating the data obtained in positive and negative samples. In this study, there are at least two possible reasons for the low detection rate of NoVs. First, it may be attributed to the steady management and preventative measures enacted throughout the ongoing investigation, such as periodic cleaning of water tanks. Second, it is likely that the samples tested were not untreated groundwater, but were instead collected from groundwater treatment systems, which perform a process that is quite similar to NoV removal. Most of the treated groundwater in South Korea is kept in a storage tank. 

The detection rate of NoV was shown to have a seasonal characteristic; only one sample isolated in winter was NoV-positive, while six out of seven NoV-positive samples were detected in summer. This may be because rainfall causes sewer overflows in summer in South Korea.

During school excursions, NoV was isolated in a waterborne outbreak in Jeju Island, South Korea. The NoVs belonged to the GI-1, GI-3, GI-4 GII-3, GII-4, GII-5, GII-6, GII-8, and GII-14 genotypes [7]. KFDA (Korean Food & Drug Administration) in 2010 reported that the GII-17 Katrina strain was the most prevalent in the three year period 2004–2006. In addition, the report revealed that the major GII genotypes in Han liver and Wang-suk River were GII-4 and GII-17 genotypes. The NoV GII genotypes that were isolated in this study detected in South Korea were as reported in previous studies [29].

A previous survey reported the NoV detection rate in untreated groundwater in South Korea as 39% (117/300) [30]. Additionally, in Singapore, the NoV detection rate in surface water was demonstrated to be 71.7% (43/60) [31]. However, in this study, the detection rate of NoV in groundwater was 0.64% (7/1,090) in treated water. It has been shown that the NoV concentration obtained in our study was not different from the concentration obtained by other methods. The sequences of the primers used in this and previous studies [30,31] differed, but those used in our study were designated to a common region and not easily replicated. Therefore, it is not likely that the primers affected the detection rate of NoV and the difference in the NoV detection rates between studies simply indicates that the water samples differed.

Food poisoning cases continue to arise [32]; therefore, preventative measures should be taken against NoV contamination of groundwater. Although this study examined NoV contamination of drinking water, further studies are required to investigate enteric virus contamination of food.
